# KPNA6 is a Cofactor of ANP32A/B in Supporting Influenza Virus Polymerase Activity

**DOI:** 10.1128/spectrum.02073-21

**Published:** 2022-01-19

**Authors:** Mengmeng Yu, Liuke Sun, Zhenyu Zhang, Yuan Zhang, Haili Zhang, Lei Na, Xiaojun Wang

**Affiliations:** a State Key Laboratory of Veterinary Biotechnology, Harbin Veterinary Research Institute, The Chinese Academy of Agricultural Sciences, Harbin, China; Changchun Veterinary Research Institute

**Keywords:** vRNP, ANP32A, ANP32B, KPNA6, influenza, polymerase

## Abstract

Influenza A virus (IAV) RNA-dependent RNA polymerase (vPol) is a heterotrimer composed of PB2, PB1, and PA, which, together with vRNA and nucleoprotein (NP), forms viral ribonucleoprotein (vRNP) complex to direct the transcription and replication of the viral genome. Host factor ANP32 proteins have been proved to be associated with vRNP and are essential for polymerase activity and cross-species restriction of avian influenza virus. However, the molecular mechanism by which ANP32 supports polymerase activity is largely unknown. Here, we identified that KPNA6 is associated with ANP32A/B and vRNP of the influenza virus. Both knockout and overexpression of KPNA6 downregulate the replication of the influenza virus by inhibiting the polymerase activity, indicating that a certain level of KPNA6 is beneficial for efficient replication of the influenza virus. Furthermore, we demonstrate that overexpression of KPNA6 or its nuclear importing domain negative mutation inhibited the interaction between ANP32 and vRNP, thus reducing the polymerase activity. Our results revealed the role of KPNA6 in interacting with both ANP32A/B and vRNP to maintain viral polymerase activity and provided new insights for further understanding of the mechanism by which ANP32 supports influenza polymerase.

**IMPORTANCE** Host factor ANP32 plays a fundamental role in supporting the polymerase activity of influenza viruses, but the underlying mechanism is largely unknown. Here, we propose that KPNA6 is involved in the function of ANP32A/B to support influenza virus polymerase by interacting with both vRNP and ANP32A/B. The proper amount of KPNA6 and ANP32 proteins in the KPNA6-ANP32-vRNP complex is crucial for maintaining the viral polymerase activity. The KPNA6 may contribute to maintaining stable interaction between vRNA and ANP32 proteins in the nucleus, and this function is independent of the known importing domain of KPNA6. Our research reveals a role of KNPA6 associated with ANP32 proteins that support the viral polymerase and suggests a new perspective for developing antiviral strategies.

## INTRODUCTION

RNA polymerase of influenza virus is a heterotrimer consisting of PB1, PB2, and PA, assembled with viral RNA and nucleocapsid protein (NP) to form a viral ribonucleoprotein (vRNP) complex to direct the transcription and replication of the viral genome (1–3). Research on the interplay between host factors and influenza virus polymerase helps us understand the replication mechanism of influenza virus RNA polymerase and develop new antiviral approaches. Many host factors regulate virus replication at different stages of the virus life cycle (4). Among them, host factor ANP32 proteins are crucial in supporting viral polymerase activity of the influenza A and B viruses and determining the interspecies restriction of the avian influenza A virus (5–12).

ANP32A from the avian host has a special 33-amino acid insertion, enabling avian ANP32A to support the polymerase activity of the avian influenza virus uniquely (5). A hydrophobic SUMO interaction motif (SIM)-like sequence within the 33-amino acid insertion is essential for its support of avian-origin polymerase (5, 13). However, mammalian ANP32A and B can support the polymerase activity only of human-adapted influenza virus and cannot efficiently support the polymerase activity of avian influenza virus, which explains the restriction of avian-origin polymerases in mammalian cells (5–7).

Others and our lab found that the polymerase activity of the influenza virus was completely lost without ANP32A and ANP32B, revealing that mammalian ANP32A and B are essential key host proteins for influenza virus replication, and both of them are functionally redundant in their support for influenza virus polymerase (6, 7, 14). Swine ANP32A has a unique amino acid evolution key site, which can support the polymerase activity of the avian influenza virus to a certain extent and enable swine to become a “mixing vessel” for influenza viruses (15, 16). In addition, we also found that avian ANP32 members cannot efficiently support the activity of influenza B virus polymerase, which might explain why avians are rarely naturally infected with influenza B viruses (11). The latest structural studies have shown that the N-terminal leucine-rich repeat structure of ANP32A bridges two asymmetric viral polymerases and mediates the assembly of the influenza virus replicase, which provides a structural basis for understanding why ANP32A could support the polymerase activity of the influenza virus (17).

Members of the importin-α family actively import vRNPs into the nucleus through the nuclear pore. Importin-α, also known as karyopherin (KPNA), was identified as an adaptor protein linking nuclear localization signal (NLS)-containing proteins with importin-β (18–20). KPNA family members are components of the classical import pathway and act as adaptors recognizing cargo proteins with a NLS. Importin-α/cargo protein complexes facilitate binding to the importin-β1 receptor protein (18). Thus, cargo proteins are transported into the nucleus as ternary complexes. KPNA proteins have been strongly implicated in influenza A host-specific adaptation, evidenced by the observation that a switch from KPNA3 to KPNA6 dependency occurs upon mammalian adaptation (21, 22). In addition, KPNA may have a role in supporting viral replication within the nucleus. Studies have shown that KPNA proteins may regulate influenza virus activity through PB2 701N or NP 319K site or recruiting other host factors to complete the transport of cargo protein through the Arm domain (23). However, the mechanism of how KPNA6 proteins regulate polymerase activity remains unclear. In addition, whether KPNA6 affects the polymerase activity supported by host factor ANP32 also remains to be answered.

This study found that KPNA6 interacts with ANP32 proteins and affects the polymerase activity supported by ANP32. The proper level of KPNA6 is crucial for efficient viral replication. Disturbance of the balance of the vRNP-ANP32-KPNA6 complex impaired viral polymerase activity. We further revealed that, in addition to the nuclear importing mechanism, KPNA6 might play an important role in maintaining the interaction between vRNP and ANP32. These results indicate an important role of KNPA6 in the molecular mechanisms by which ANP32 proteins support polymerase activity.

## RESULTS

### KPNA6 is associated with the vRNP-ANP32A complex and could inhibit the polymerase activity of the influenza virus when overexpressed.

**FIG 1:**
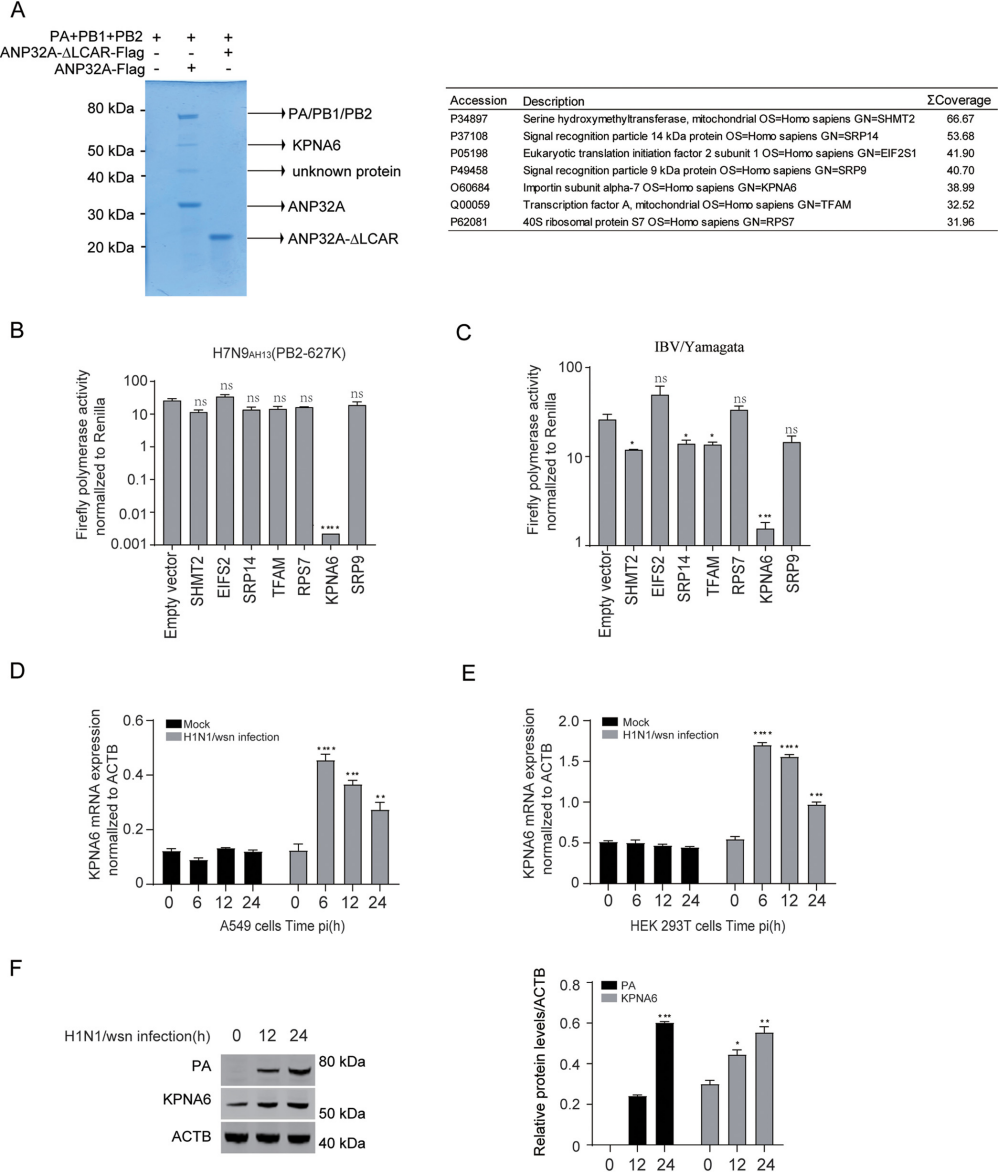
Overexpression of KPNA6 inhibits the polymerase activity of influenza virus. (A) Flag-tagged ANP32A constructs or the low-complexity acidic region (LCAR 162 to 249) truncations of ANP32A (ANP32A-ΔLCAR), together with PB1, PB2, and PA expression plasmids of H7N9_AH13_(PB2-627K), were transfected into HEK293T cells. The immunoprecipitated samples were subjected to SDS-PAGE analysis with Coomassie blue staining following anti-Flag precipitation. The corresponding proteins are marked with an arrow (A, left); high-confidence candidates were identified by mass spectrometry (MS) analysis with the immunoprecipitated samples and are shown as a table (A, right). (B and C) Minigenome assay of H7N9_AH13_(PB2-627K) (B) and IBV-Yamagata (C) in human HEK293T cells with coexpressed empty vector and different Flag-tagged constructs. Error bars represent the standard deviation (SD) of the replicates within one representative experiment. NS, not significant; *, *P *<* *0.05; ***, *P *<* *0.001; ****, *P *<* *0.0001. (D and E) The mRNA level of endogenous KPNA6 in A549 (D) and HEK293T (E) cells in response to IAV (H1N1_WSN_) infection (MOI = 0.01). The cells were collected at 0, 12, 24, 36, and 48 h postinfection, followed by quantitative PCR to measure the mRNA level of KPNA6. Error bars represent the SD of the replicates within one representative experiment. NS, not significant; *, *P *<* *0.05; ***, *P *<* *0.001; ****, *P *< 0.0001. (F) The protein level of endogenous KPNA6 in HEK293T cells in response to IAV (H1N1_WSN_) infection (MOI = 0.01). The cells were collected at 0, 12, and 24 h postinfection, followed by Western blotting of KPNA6 and influenza polymerase subunit PA. The bar represents the levels of KPNA6 and PA in the left panel, which were normalized to ACTB. NS, not significant; *, *P *<* *0.05; **, *P *<* *0.01; ***, *P *<* *0.001.

Host factor ANP32A is essential for influenza virus replication and is associated with the viral polymerase trimeric complexes (17). To illustrate whether other host factors are involved in the function of ANP32 in supporting influenza virus polymerase, we purified the viral polymerase trimeric complexes by coimmunoprecipitation with ANP32A to screen the potential candidates that interact with viral polymerase trimeric complexes or ANP32A. The low-complexity acidic region (LCAR; 162 to 249) truncations of human ANP32A (ANP32A-ΔLCAR) that lost the ability to bind with the viral polymerase trimeric complexes were used as a negative control in our immunoprecipitation experiments (Fig. 1A, left panel). Seven proteins with high scores in mass spectrometry analysis were selected and used for further experiments (Fig. 1A, right panel). We next overexpressed these 7 candidates together with the polymerase reporter to see if they affected the polymerase activity of influenza A and B virus in HEK293T cells. We found that overexpression of KPNA6, but not other proteins, dramatically downregulated the polymerase activity for both influenza A and B virus (Fig. 1B and C). Moreover, the mRNA level and protein level of endogenous KPNA6 in A549 and HEK293T cells increased in response to influenza A virus infection (Fig. 1D to F). These data indicated that KPNA6 regulates the polymerase activity of the influenza virus, and its expression is upregulated with influenza virus infection.

### The appropriate level of KPNA6 is important for efficient viral replication.

**FIG 2:**
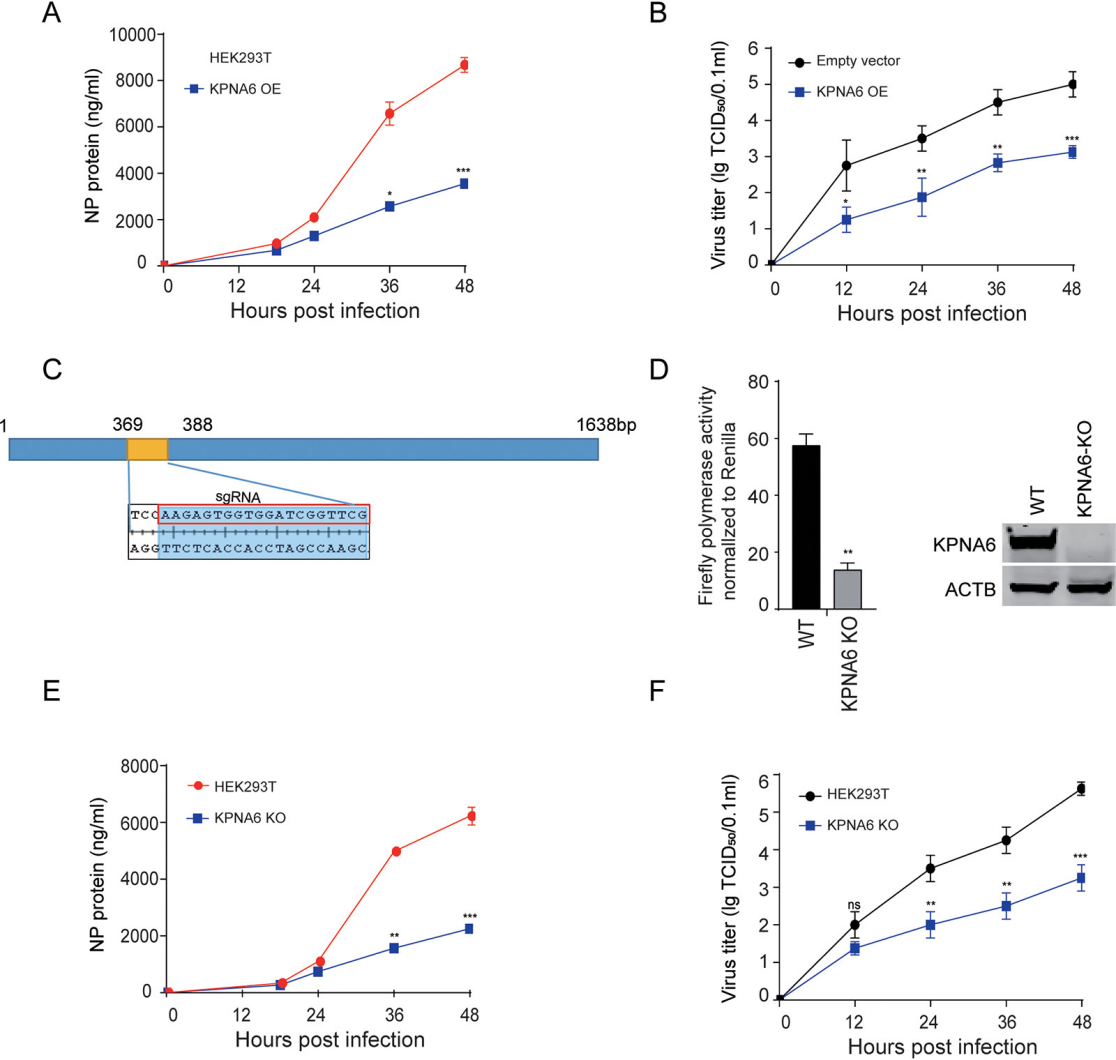
Both overexpression and KPNA6 gene knockout affect influenza virus replication. (A and B) Impact of KPNA6 overexpression on IAV replication. HEK293T cells overexpressing KPNA6 or empty control were infected with IAV (H1N1_WSN_) (MOI = 0.01), the supernatant was collected at described time points, and the NP content (A) and virus titers (B) in the supernatant were measured with AC-ELISA and 50% tissue culture infective dose (TCID_50_), respectively. Error bars represent the SD of the replicates within one representative experiment. NS, not significant; *, *P *<* *0.05; **, *P *<* *0.01; ***, *P *<* *0.001. (C) Schematic diagram of KPNA6 sgRNA target positions in its coding region. (D) (left panel) Measurement of H7N9_AH13_(PB2-627K) vPol activity in wild-type (WT) and KPNA6 knockout cell lines and (right panel) Western blot of HEK293T cells lysates from left panel. (E to F) Impact of KPNA6 loss on IAV replication. WT and KPNA6 knockout cell lines were infected with influenza A virus (H1N1_WSN_) (MOI = 0.01), the supernatant was collected at described time points, and the NP content (E) and virus titers (F) in the supernatant were measured with AC-ELISA and TCID_50_, respectively. Error bars represent the SD of the replicates within one representative experiment. NS, not significant; *, *P *<* *0.05; **, *P *<* *0.01; ***, *P *<* *0.001.

To further assess the effect of overexpression of KPNA6 on the replication of influenza A virus, we infected 293T cells overexpressing KPNA6 with H1N1_WSN_ virus (multiplicity of infection [MOI] = 0.001) and measured the NP content and virus titer in the cell supernatant over time. We found that overexpression of KPNA6 significantly inhibits virus replication (Fig. 2A and B). We next used a CRISPR/Cas9 system to knock out endogenous KPNA6 in HEK293T cells to determine the effect of KPNA6 loss on the polymerase activity and influenza A virus replication (Fig. 2C and D). We found that loss of KPNA6 impaired polymerase activity in HEK293T cells. Multicycle growth kinetics of influenza A viruses in the KPNA6 knockout cell lines suggested that KPNA6 deficiency also inhibits virus replication (Fig. 2E and F), consistent with previous KPNA6 knockdown results (22, 24). Therefore, the above data suggested that efficient polymerase activity depends on the appropriate expression level of KPNA6.

### KPNA6 affects the interaction between ANP32 and influenza virus polymerase complex.

**FIG 3:**
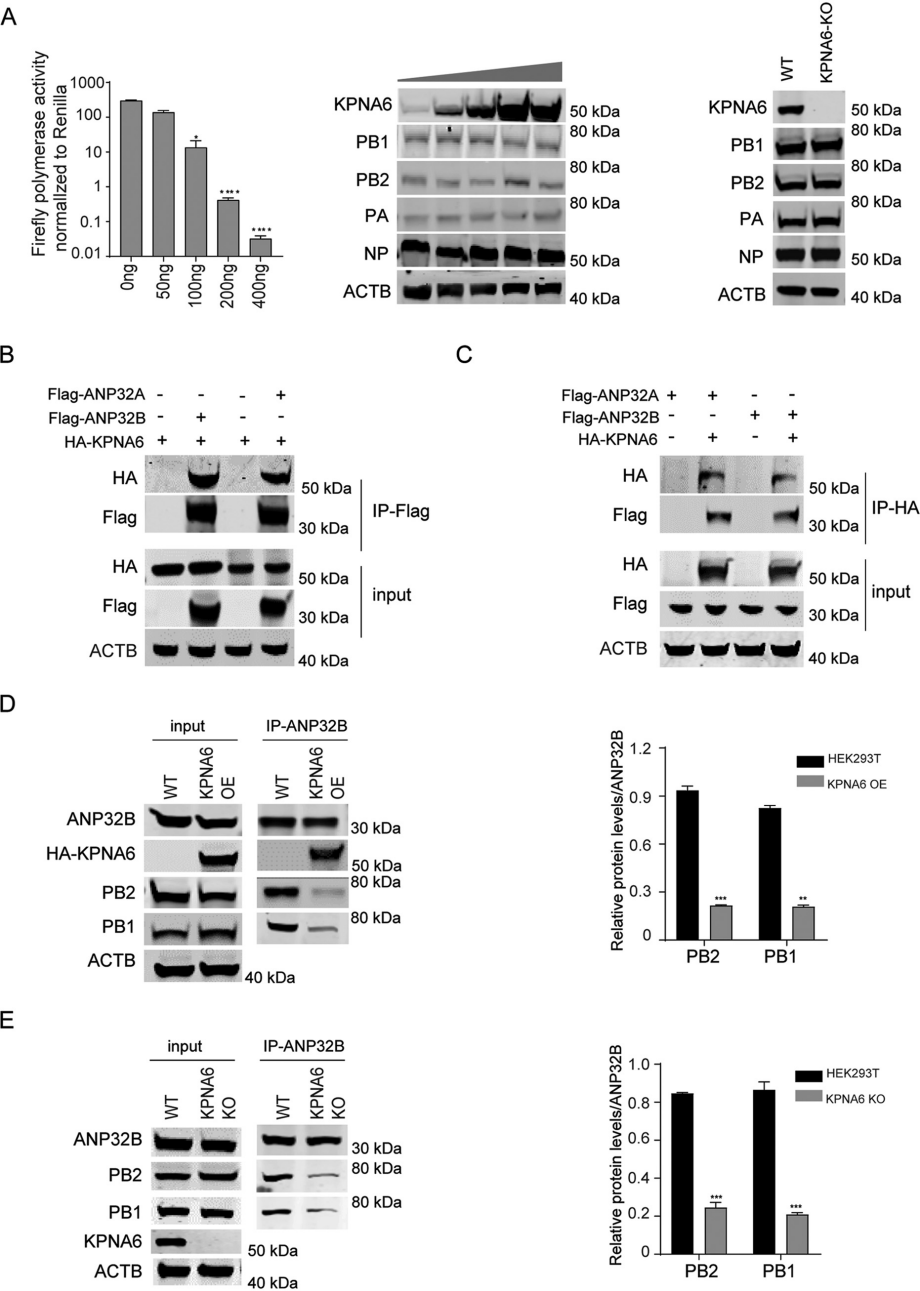
KPNA6 affects ANP32-vRNP interaction. (A) (left) The dose-dependent effect of KPNA6 expression on the polymerase activity of H7N9_AH13_(PB2-627K) polymerase activity assays was conducted by transfection with indicated plasmids in HEK293T cells, (middle) Western blot of 293T cell lysates from the left panel, and (right) Western blot of polymerase protein expression in WT and KPNA6 knockout cell lines. Error bars represent the SD of the replicates within one representative experiment. *, *P *<* *0.05; ****, *P *<* *0.0001. (B and C) 293T cells were transfected with either empty control or the indicated Flag-tagged constructs, together with HA-tagged KPNA6. Following anti-Flag precipitation (B) and anti-HA precipitation (C), the indicated proteins were detected by Western blotting. (D) 293T cells were transfected with either empty control or HA-tagged KPNA6, together with the viral trimeric polymerase, NP, and a minigenome reporter pHH21-huPolI-vLuc. Following anti-huANP32B precipitation, the indicated proteins were detected by Western blotting. The bar graph represents the relative amount of PB1 and PB2 immunoprecipitated by the ANP32B. Error bars represent the SD of the replicates from three independent experiments. **, *P *<* *0.01; ***, *P *<* *0.001. (E) HEK293T cells and KPNA6 knockout cell lines were transfected with the viral trimeric polymerase, NP, and a minigenome reporter pHH21-huPolI-vLuc. Following anti-huANP32B precipitation, the indicated proteins were detected by Western blotting. The bar graph represents the relative amount of PB1 and PB2 immunoprecipitated by the ANP32B. Error bars represent the SD of the results from three independent experiments. ***, *P *<* *0.001.

Although KPNA6 inhibits the polymerase activity of the influenza virus in a dose-dependent manner, the expression level of the viral polymerase and NP was not affected (Fig. 3A). It has been reported that KPNA6 does not affect the assembly of influenza virus vRNP (24). Host factors ANP32A and ANP32B play an indispensable role in supporting the polymerase activity and influenza virus replication by binding with vRNP. Considering that the KPNA6 was immunoprecipitated by ANP32A, we next investigated whether the inhibiting effect of overexpression of KPNA6 on the polymerase activity is related to host factor ANP32A. We found that KPNA6 has a strong binding ability with ANP32A and ANP32B (Fig. 3B and C). Overexpression of KPNA6 inhibited the binding of ANP32B to the influenza virus polymerase complex (Fig. 3D).

**FIG 4:**
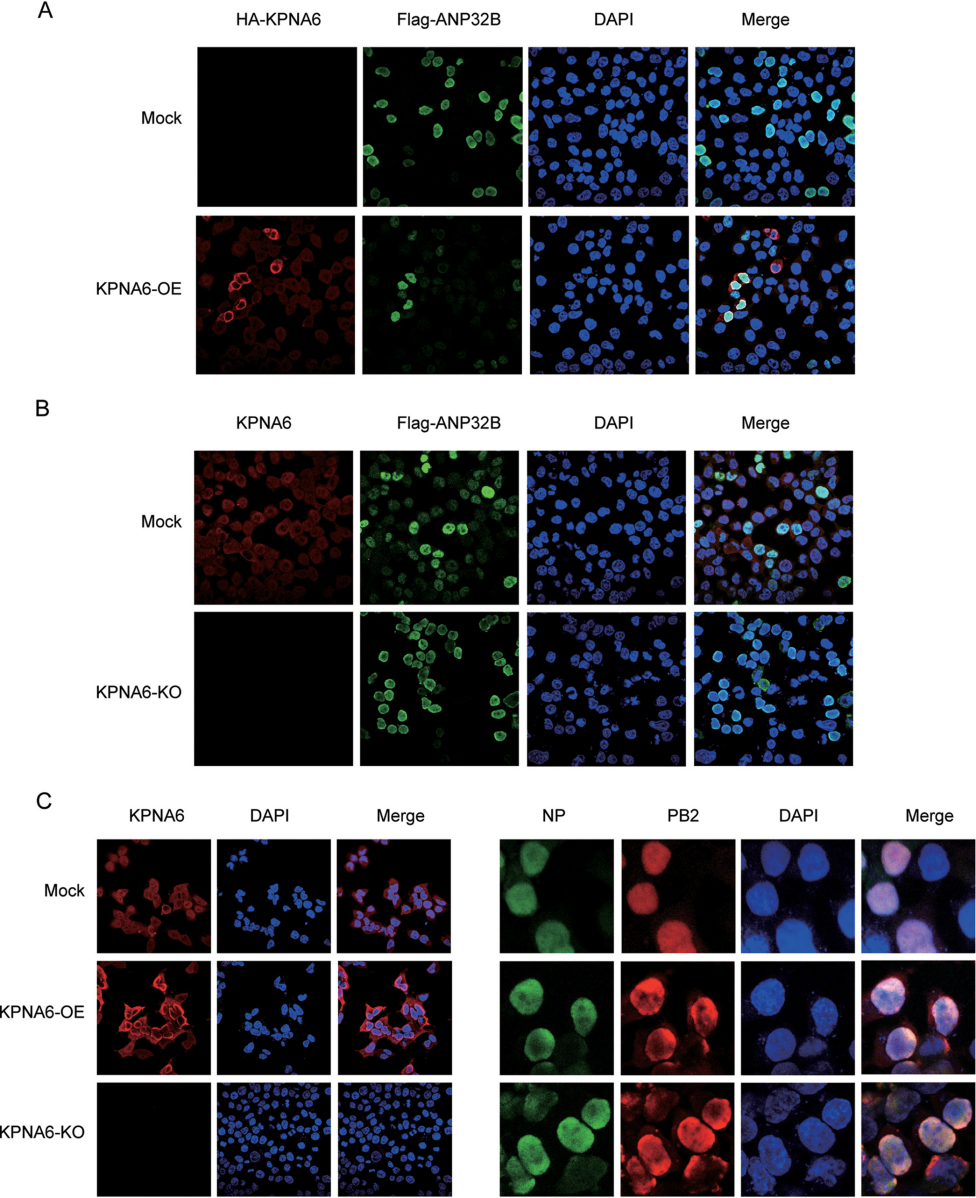
KPNA6 knockout or overexpression does not affect the localization of ANP32 and vRNP. (A) Immunofluorescence analysis of the indicated HA-KPNA6 and Flag-ANP32B in HEK293T cells. (B) Immunofluorescence analysis of the indicated KPNA6 and Flag-ANP32B in HEK293T and KPNA6-KO cells. (C) Immunofluorescence analysis of the indicated KPNA6, PB2, and NP in HEK293T and KPNA6-KO cells.

Furthermore, KPNA6 loss also inhibited the binding of ANP32B to the vRNP (Fig. 3E). These data suggested that KPNA6 interacts with ANP32 and vRNP. The inappropriate expression of KPNA6 could disturb viral polymerase activity by weakening the ANP32-vRNA interaction. The main physiological function of KPNA6 is known to participate in the transport of nuclear proteins. Our results show that overexpression or knockout of KPNA6 did not affect the nuclear location of ANP32 proteins (Fig. 4A and B) and vRNP (Fig. 4C), indicating that the inhibition effect on the polymerase activity exerted by KPNA6 was not caused by the changes in the location of ANP32 proteins and vRNP.

**FIG 5:**
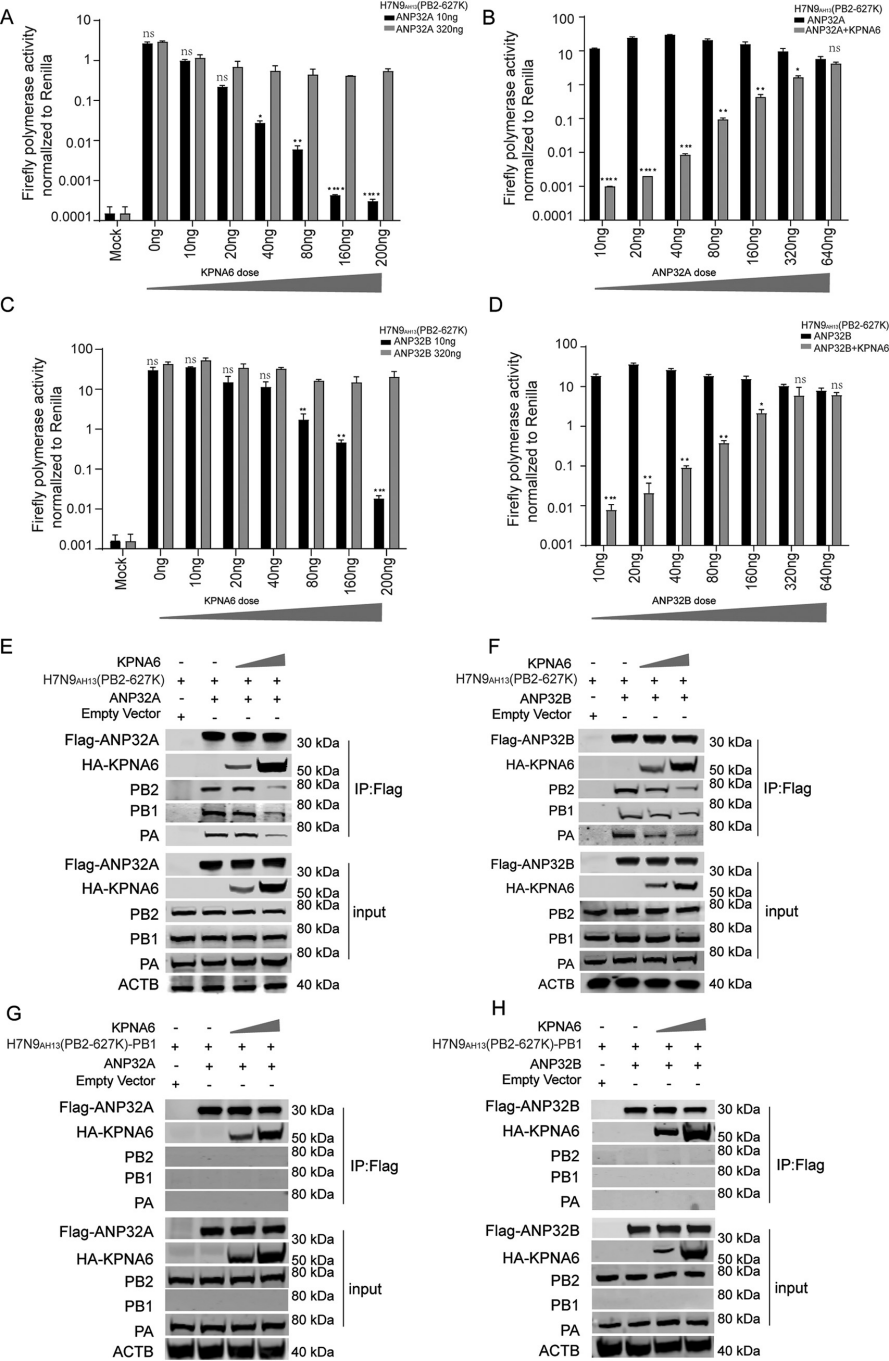
KPNA6 inhibits ANP32-vRNP interaction in a dose-dependent manner. (A) The effect of the increased amount of KPNA6 overexpression on the 10 or 320 ng of ANP32A and ANP32B supported H7N9_AH13_(PB2-627K) vPol activity in DKO cells. Error bars represent the SD of the replicates within one representative experiment. NS, not significant; *, *P *<* *0.05; **, *P *<* *0.01; ****, *P *<* *0.0001. (B) DKO cells were transfected with the H7N9_AH13_(PB2-627K) minigenome reporting system and 200 ng of KPNA6 and increased ANP32A. Luciferase activity was measured 24 h after transfection. Error bars represent the SD of the replicates within one representative experiment. NS, not significant; *, *P *<* *0.05; **, *P *<* *0.01; ***, *P *<* *0.001; ****, *P *<* *0.0001. (C) The effect of the increased amount of KPNA6 overexpression on the 10 ng or 320 ng of ANP32B supported H7N9_AH13_(PB2-627K) vPol activity in DKO cells. Error bars represent the SD of the replicates within one representative experiment. NS, not significant; **, *P *<* *0.01; ***, *P *<* *0.001. (D) DKO cells were transfected with the H7N9_AH13_(PB2-627K) minigenome reporting system and 200 ng of KPNA6 and increased ANP32B. Luciferase activity was measured 24 h after transfection. Error bars represent the SD of the replicates within one representative experiment. NS, not significant; *, *P *<* *0.05; **, *P *<* *0.01; ***, *P *<* *0.001. (E) The impact of the increased amount of ectopic expression of KPNA6 on the ANP32A-vRNP interactions. An experiment was performed as in Fig. 3D. (F) The impact of the increased amount of ectopic expression of KPNA6 on the ANP32B-vRNP interactions. An experiment was performed as in Fig. 3D. (G) Experiment performed as in panel E, except the PB1 was omitted when transfected to prove that ANP32A binds only to vRNP. (H) Experiment performed as in panel F, except the PB1 is omitted when transfected to prove that ANP32B binds only to vRNP.

To further prove that the appropriate level of KPNA6 is important for ANP32 to support influenza polymerase activity and to bind to vRNP, ANP32A and ANP32B double gene knockout cell lines (DKO) (6) were used to quantitatively control the expression levels of ANP32 and KPNA6 for polymerase activity determination. Our results show that when there is a low dose of ANP32A or ANP32B (10 ng/well), overexpression of KPNA6 inhibits the polymerase activity in a dose-dependent manner (Fig. 5A and C). However, overexpression of ANP32A or ANP32B (320 ng/well) could counteract the inhibition effect of influenza virus polymerase caused by overexpression of KPNA6 (Fig. 5A and C). Similarly, the inhibition of ANP32A/B-mediated polymerase activity by a certain amount of KPNA6 in DKO cells could be reversed by overexpression of ANP32A or ANP32B in a dose-dependent manner (Fig. 5B and D). Some of the cell lysates from Fig. 5A and C were used in the coimmunoprecipitation (co-IP) experiment, which clearly showed that along with the expression of KPNA6 being increased, the vRNP bound by ANP32A/B was reduced (Fig. 5E and F). However, when the viral polymerase subunit PB1 was absent, no PB2 and PA coimmunoprecipitated with ANP32 and KPNA6, indicating that ANP32A or ANP32B binds to vRNP but not to a single subunit (Fig. 5G and H). Overall, the above results confirm that the appropriate level of KPNA6 helps ANP32 bind to vRNP and support the influenza virus’s polymerase activity efficiently.

### The interaction between KPNA6 and ANP32A/B is involved in KPNA6’s regulation of influenza virus polymerase activity supported by ANP32A/B.

**FIG 6:**
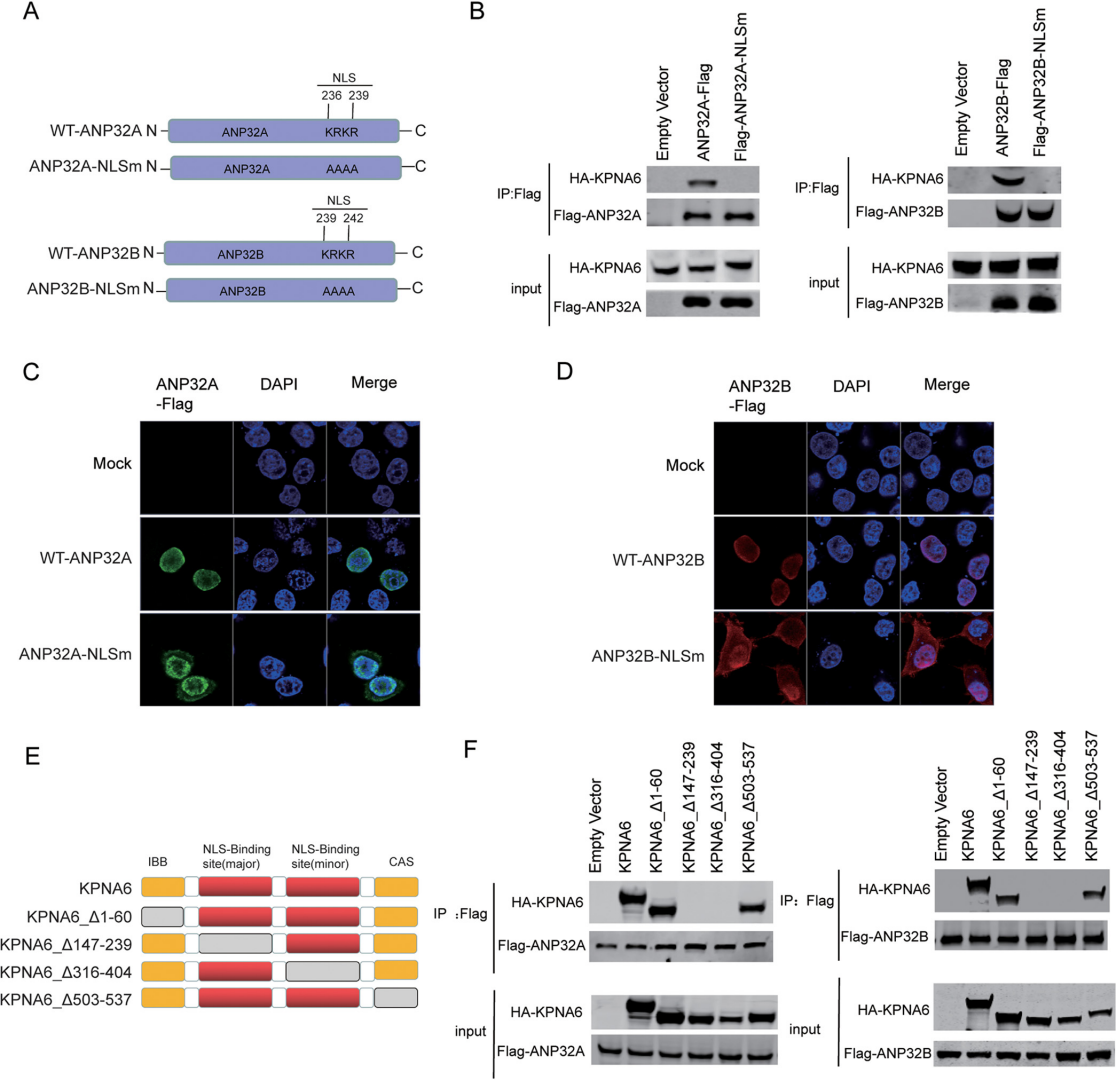
Identification of key domains involved in the interaction of ANP32 and KPNA6. (A) Cartoon showing the wild type of human ANP32 proteins and the mutants (ANP32A-NLSm) with destroyed nuclear location signal by mutating KRKR into AAAA. The NLS area of ANP32A is 236 to 239, and the NLS of ANP32B is 239 to 242. (B) ANP32A-Flag and ANP32A-NLSm-Flag were cotransfected with KPNA6-HA into HEK293T cells. Following anti-Flag precipitation 24 h after transfection, the immunoprecipitated samples were subjected to Western blotting (left panel). Experiments were performed in the left panel, except that ANP32B-Flag and ANP32B-NLSm-Flag were used for transfection (right panel). (C and D) Immunofluorescence analysis of the indicated Flag constructs in HEK293T cells. (E) Cartoon showing the structure of the different KPNA6 truncations and the position of different key domains in KPNA6, which includes an N-terminal importin β-binding (IBB) domain, armadillo (Arm) repeats that function as internal cargo NLS-binding sites, and a C-terminal region (CAS). (F) Different HA-tagged KPNA6 truncations and Flag-tagged ANP32A (left panel) or Flag-tagged ANP32B (right panel) were transfected into HEK293T cells. Following anti-Flag precipitation 24 h after transfection, the immunoprecipitated samples were subjected to Western blotting.

To determine the molecular mechanism by which overexpression of KPNA6 inhibits the interaction between ANP32 and the influenza virus polymerase complex, we first determined the key domains of the interaction between KPNA6 and ANP32A or ANP32B. KPNA6 completes the transport of cargo protein through the Arm domain, which serves as the internal cargo NLS-binding site (25). Both ANP32A and ANP32B have a nuclear localization signal, a key domain for binding KPNA6. Previous study has also suggested that ANP32 may bind to the KPNA family (26). To confirm this hypothesis, we mutated the nuclear localization signal KRKR of ANP32A/B to AAAA (ANP32-NLSm) (Fig. 6A), and results show that this mutation caused the loss of interaction between ANP32 and KPNA6 (Fig. 6B). At the same time, we also found that mutations in the nuclear localization signal sequence can only slightly inhibit the ANP32A/B protein from entering the nucleus (Fig. 6C and D). On the other hand, a series of constructs of KPNA6 were made according to the domains of KPNA6 (Fig. 6E). The results showed that two constructs of KPNA6 (Δ147–239 or Δ316–404), which lacks the nuclear localization signal binding region, impaired its interaction with ANP32A/B (Fig. 6F).

**FIG 7:**
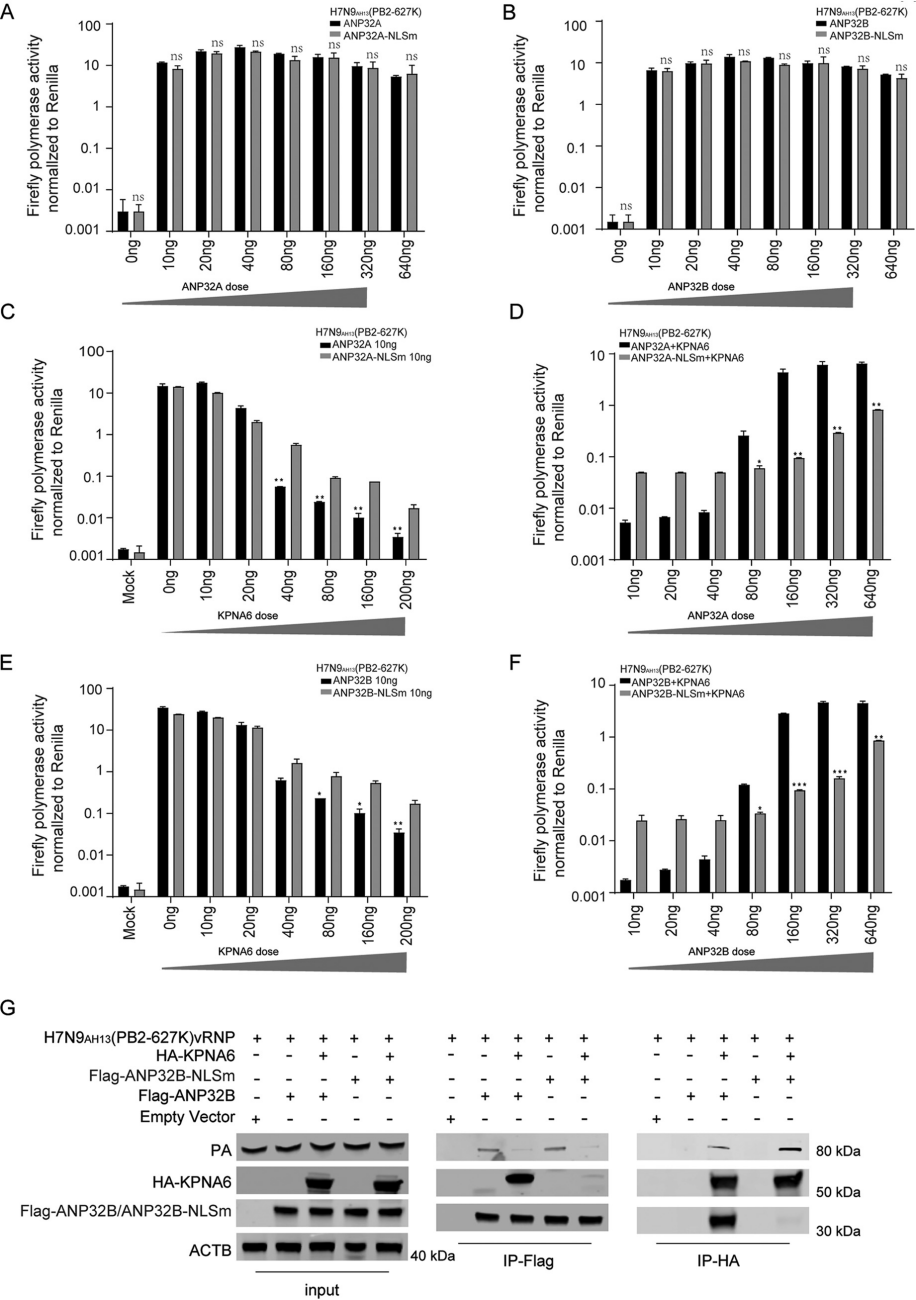
KPNA6 competitively binding vRNP with ANP32A to inhibit polymerase activity supported by ANP32 proteins. (A) DKO cells were transfected with H7N9_AH13_(PB2-627K) minigenome reporting system, with an increased amount of either ANP32A or ANP32A-NLSm. Luciferase activity was analyzed as mentioned above. Error bars represent the SD of the replicates within one representative experiment. NS, not significant. (B) DKO cells were transfected with H7N9_AH13_(PB2-627K) minigenome reporting system, with an increased amount of either ANP32B or ANP32B-NLSm. Luciferase activity was analyzed as mentioned above. Error bars represent the SD of the replicates within one representative experiment. NS, not significant. (C) The impact of an increased amount of KPNA6 overexpression on the H7N9_AH13_(PB2-627K) vPol activity supported by ANP32A or ANP32A-NLSm. Error bars represent the SD of the replicates within one representative experiment. **, *P *<* *0.01. (D) DKO cells were transfected with the H7N9_AH13_(PB2-627K) minigenome reporting system together with 200 ng KPNA6 and an increased amount of ANP32A or ANP32A-NLSm. Luciferase activity was measured 24 h after transfection. Error bars represent the SD of the replicates within one representative experiment. *, *P *<* *0.05; **, *P *<* *0.01. (E and F) Experiments performed as in panels C and D, respectively, except that ANP32B and ANP32B-NLSm were used. (G) DKO cells were transfected with the H7N9_AH13_(PB2-627K) minigenome reporting system and different constructs as indicated in the figure. Twenty-four hours after transfection, cell lysates were used for anti-Flag precipitation and anti-HA precipitation. The immunoprecipitated samples were subjected to Western blotting.

To assess the effect of the interaction of KPNA6 and ANP32 on the polymerase activity of influenza virus supported by ANP32, reconstitution of either ANP32 or ANP32-NLSm in DKO was performed when KPNA6 was overexpressed in the polymerase activity assay. We found that ANP32-NLSm still retains the ability to support influenza virus polymerase activity (Fig. 7A and B). However, KPNA6 overexpression has significant differences in the inhibitory effects of ANP32 and ANP32-NLS mutants-mediated polymerase activity (Fig. 7C and E), and overexpression of ANP32 and that of ANP32-NLSm have significant differences in the antagonistic effect on the inhibition of influenza virus polymerase by overexpression of KPNA6 (Fig. 7D and F). Overall, these data indicate that the interaction between KPNA6 and ANP32 is important for KPNA6 to regulate the polymerase activity supported by ANP32.

### The interaction between KPNA6 and vRNP is involved in KPNA6’s regulation of influenza virus polymerase activity supported by ANP32.

Even though the inhibitory effects of overexpression of KPNA6 on ANP32 and ANP32-NLSm are significantly different, KPNA6 could still dramatically inhibit the polymerase activity supported by ANP32-NLSm (Fig. 7C and E). At the same time, compared with ANP32, increasing the dose of ANP32-NLS mutant expression can only partially antagonize the inhibitory effect of KNPA6 on ANP32-mediated polymerase activity (Fig. 7D to F), which suggests that apart from interacting with ANP32, there are other molecular mechanisms involved in KPNA6’s function to regulate influenza virus polymerase activity. Considering its binding to vRNP, KPNA6 inhibits polymerase activity by binding vRNP competitively with ANP32. Co-IP experiments were performed to verify this hypothesis, and the results showed that in the presence of ANP32 (Fig. 7G), the amount of KPNA6 bound to vRNP was significantly smaller than that of ANP32-NLSm (Fig. 7G, IP-HA), which explains the reason responsible for the significant difference (Fig. 7D to F). Overexpression of KPNA6 reduced the activity of polymerases supported by wild-type ANP32 and ANP32-NLSm and ultimately led to a decrease in their interaction with vRNP (Fig. 7G, IP-Flag). Overall, these data indicate that competitively binding vRNP with ANP32A is another mechanism adopted by KPNA6 to inhibit ANP32-mediated influenza virus polymerase activity.

**FIG 8:**
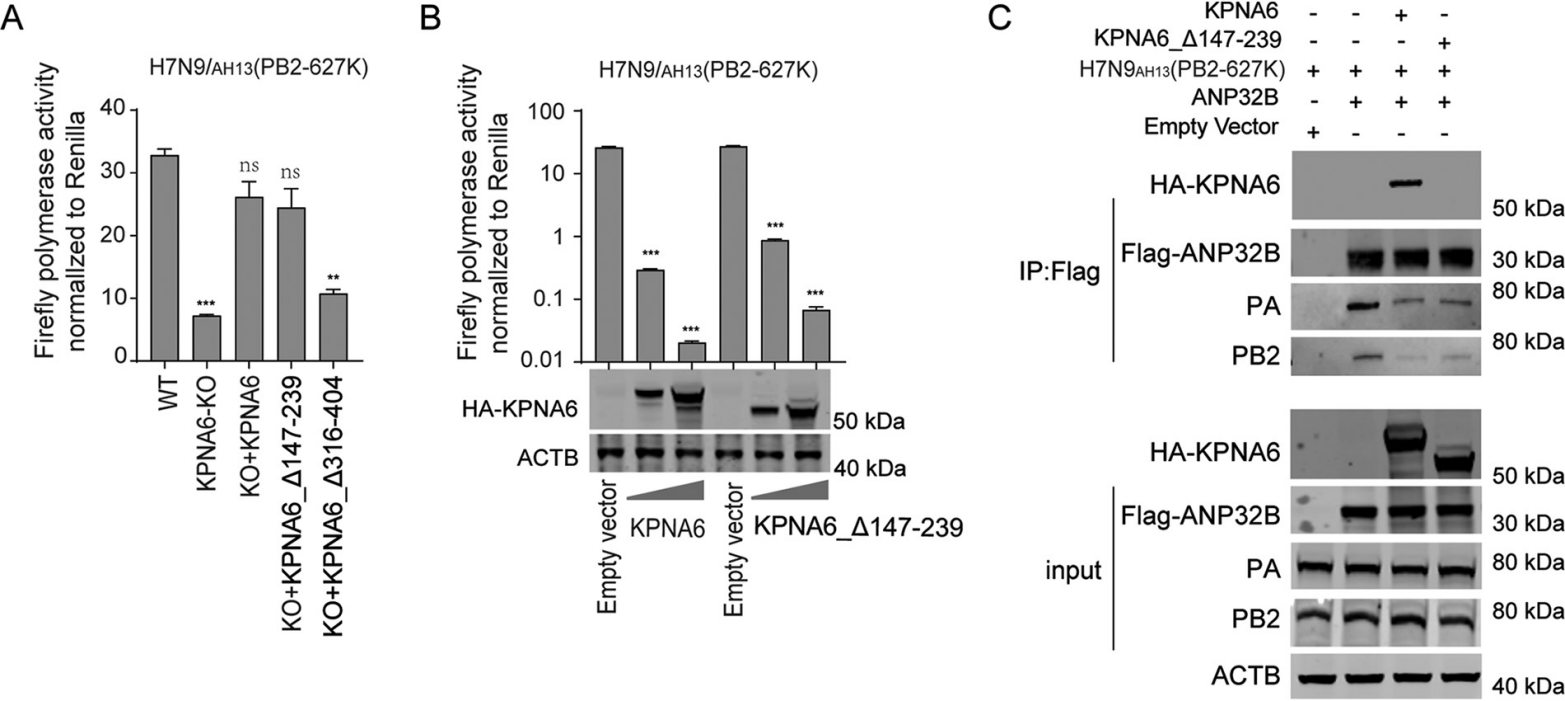
KPNA6 without NLS domain (KPNA6-Δ147–239) can still inhibit polymerase activity. (A) HEK293T and KPNA6-KO cells were transfected with the H7N9_AH13_(PB2-627K) minigenome reporting system and different constructs, KPNA6, KPNA6-Δ147–239, KPNA6-Δ316–404. Luciferase activity was measured 24 h after transfection. Error bars represent the SD of the replicates within one representative experiment. NS, not significant; **, *P *<* *0.01; ***, *P *<* *0.001. (B) DKO cells were transfected with the H7N9_AH13_(PB2-627K) minigenome reporting system and ANP32B, KPNA6, KPNA6-Δ147–239. Luciferase activity was measured 24 h after transfection. The samples were subjected to Western blotting. Error bars represent the SD of the replicates within one representative experiment. ***, *P *<* *0.001. (C) DKO cells were transfected with the H7N9_AH13_(PB2-627K) and different constructs, as indicated in the figure. Twenty-four hours after transfection, cell lysates were used for anti-Flag precipitation. The immunoprecipitated samples were subjected to Western blotting.

The above research results indicated that KPNA6 interacts with ANP32A/B and viral vRNP and affects the polymerase activity. Considering that KPNA6 will bind to ANP32 and other proteins containing NLS sequence, overexpression of KPNA6 may inhibit polymerase activity by sequestering natural binding partners like ANP32 or vRNP. To test whether other molecular mechanisms were involved in the antiviral function of KPNA6, we used the KPNA6-Δ147–239 mutant, which lost the major binding ability to the NLS domain of target proteins like ANP32, to perform the following experiments. As shown in Fig. 8A, reconstitution of either KPNA6 or KPNA6-Δ147–239 mutant in the KPNA6 knockout cells increased the polymerase activity of H7N9_AH13_(PB2-627K) (Fig. 8A). Moreover, in the case of overexpression, both KPNA6 and KPNA6-Δ147-239 mutant could inhibit the polymerase activity of H7N9_AH13_(PB2-627K) (Fig. 8B) in a dose-dependent manner. The co-IP experiment further proved that KPNA6-Δ147–239 mutant significantly inhibited the interaction between ANP32 protein and vRNP (Fig. 8C). These results indicated that apart from its nuclear importing role, KPNA6 could disturb the interaction between ANP32 and vRNP, influencing the viral replication.

## DISCUSSION

Research on the interplay between the host factors and the replication machine of influenza viruses has always been a hot spot in the field of influenza virus research (4, 27). ANP32A and B have been proven to be fundamental host factors for influenza virus replication and play a decisive role in replication and cross-species transmission of avian influenza virus (4–6). Although it was shown that ANP32A/B bind to vRNP of influenza virus and affect the production of influenza virus cRNA and vRNA (6, 7, 17), the specific mechanism underlying the polymerase activity supported by ANP32 is not clear. Therefore, we discovered that KPNA6 is one of the associated proteins of ANP32A and affects its interaction with vRNP. KPNA6 can bind to both ANP32A/B and vRNP, affecting influenza A virus replication by regulating the interaction between ANP32 and the vRNP of influenza A virus, indicating a role of KPNA6 in the interplay between host factor ANP32 and the vRNP of influenza A virus.

In early studies, it was reported that KPNA6 knockdown affects the replication of the influenza A virus (22, 24) and KPNA6 may regulate influenza virus replication by interacting with an unknown key host factor in the nucleus (24, 28), but the underlying mechanism has not been elucidated. Our research made a similar observation when we knocked out endogenous KPNA6 in HEK293T cells. Although KPNA6 knockdown does not affect the location and expression level of the polymerase subunits or the assembly of the vRNP (24), it inhibits the influenza virus polymerase activity. We speculated that the inhibiting effect of overexpression of KPNA6 on the polymerase activity might indirectly function through ANP32. Most importantly, we found that overexpression of KPNA6 inhibits viral polymerase activity dramatically without affecting the expression level of polymerase subunits. Therefore, our experimental results prove that KPNA6 affects virus replication, largely due to the interaction between ANP32 and vRNP. We conclude that an appropriate level of KPNA6 is very important for efficient replication of the influenza A virus and that both knockout and overexpression of KPNA6 are harmful to virus replication.

It is worth noting that the level of endogenous KPNA6 was upregulated with influenza A virus infection, suggesting that KPNA6 may play a certain regulatory role in the process of virus replication. Therefore, it is particularly important to explore its regulatory mechanism for influenza viruses. We found that apart from binding to vRNP, which is consistent with an earlier report (24), KPNA6 directly interacts with host factor ANP32 mediated by the NLS-binding site of KPNA6 and the NLS region of ANP32. To evaluate whether the effect of overexpression of KPNA6 on polymerase activity is related to ANP32, we performed polymerase activity assay in ANP32A/B double knockout cell line (DKO) to exclude the interference of endogenous ANP32A/B and found that overexpression of KPNA6 indeed dramatically inhibited ANP32A/B-mediated polymerase activity of influenza A virus.

Binding to vRNP of influenza virus is required for host factor ANP32 in supporting polymerase activity. Because KPNA6 binds to both vRNP and ANP32, we constructed a variant of ANP32 (ANP32-NLSm) that does not bind to KPNA6 to evaluate the role of ANP32-KPNA6 interaction in the inhibitory activity of overexpression of KPNA6. We found that the inhibitory effect of KPNA6 on the polymerase activity supported by ANP32-NLS was significantly lower than that of wild-type ANP32 but still had a strong inhibitory effect, indicating that KPNA6 affects influenza virus polymerase activity through two distinct mechanisms. KPNA6 inhibits the interaction between the ANP32 and the vRNP by binding to the ANP32 protein through its nuclear localization signal, and KPNA6 may bind to the vRNP competitively with ANP32.

Furthermore, we provided evidence that in the presence of wild-type ANP32, the amount of KPNA6 bound to vRNP was much smaller than that in the presence of ANP32-NLSm. Nevertheless, in either case, overexpression of KPNA6 inhibited the interaction between ANP32 and the vRNP of influenza A virus, thereby reducing ANP32-supported polymerase activity. Interestingly, we found that KPNA6 lacking the NLS-binding region can still regulate influenza virus polymerase activity by disturbing the interaction between ANP32B and viral polymerase, which further illustrates the role of KPNA6 in maintaining the viral polymerase activity mechanism of KPNA6.

KPNA6 (importin-α 7) is a typical interspecies restriction factor. Adapting from avians to mammalian species changes the dependence of importin-α from importin-α 3 to importin-α 7 (22, 23). Like importin-α 7, ANP32A/B is also a typical interspecies restriction factor identified in recent years (5). Compared with human ANP32A, avian ANP32A has an additional 33 amino acid inserts, which makes them specific in supporting the polymerase activity of the avian influenza virus. Most ANP32A and B from different species can support viral polymerase activity, but chANP32B is naturally inactive in supporting polymerase activity due to mutations at sites 129 and 130 (6, 7). Therefore, the relationship between ANP32A/B and KPNA6 in species restriction is worthy of further study.

In summary, we found that KPNA6 regulates polymerase activity of influenza A virus supported by ANP32. The excessive or low expression level of KPNA6 affects the polymerase and viral replication activity. Both KPNA6-vRNP and KPNA6-ANP32 interactions inhibit the binding of vRNP to the ANP32, thereby inhibiting the replication of the influenza A virus. Our work contributes to understanding the mechanism by which ANP32A/B regulates influenza virus replication and provides a new target for developing new anti-influenza drugs.

## MATERIALS AND METHODS

### Cells, viruses, and plasmids.

HEK293T cells and DKO cells (ANP32A and B knockout cells from our lab) were maintained in Dulbecco modified Eagle’s medium (DMEM, Sigma) with 10% fetal bovine serum, 1% penicillin and streptomycin and kept at 37°C with 5% CO_2_. Plasmids encoding the polymerases of the influenza A virus and influenza B virus and human ANP32A/B genes were described previously (6, 11). The other pCAGGS vector plasmids encoding SHMT2 (NM_001166356.2, NP_001159828.1), EIF2S1 (NM_004094.5, NP_004085.1), SRP14 (NM_001309434.1, NP_001296363.1), TFAM (NM_001270782.2, NP_001257711.1), RPS7 (NM_001011.4, NP_001002.1), KPNA6 (NM_012316.5, NP_036448.1), and SRP9 (NM_001130440.2, NP_001123912.1), mutations of nuclear localization signal with ANP32A/B (pcAGGS- ANP32A/B-NLSm-Flag), and mutants of pcAGGS-KPNA6-Δ1-60, pcAGGS-KPNA6-Δ147-239, pcAGGS-KPNA6-Δ316-404, and pcAGGS-KPNA6-Δ503-537 were constructed based on the pCAGGS expression vector according to the online In-Fusion HD Cloning Kit User Manual.

### Knockout cell lines.

We used gRNAs design tools (http://crispor.tefor.net/) to design KPNA6 single guide RNA (sgRNA) (23). The construction method of a gene knockout cell line is the same as described previously (10). Briefly, HEK293T cells were cotransfected with 1 μg pMJ920 (from Jennifer Doudna [Addgene plasmid no. 42234]) (24) plasmid and 1 μg gRNA into one well of a 6-well plate by polyethylenimine (PEI), 24 h after transfection GFP-positive cells were sorted by fluorescence-activated cell sorting (FACS), and then monoclonal knockout cell lines were screened by Western blotting.

### Mass spectrometry analysis.

Samples were separated by SDS–PAGE and stained with Coomassie blue. Gel slices containing the proteins to be identified by mass spectrometry were treated with trypsin (Sigma) for 12 h at 37°C. The peptides were extracted from the gel slices using 0.1% trifluoroacetic acid and 50% acetonitrile and then concentrated using vacuum centrifugation. The resulting peptide solution was deionized using a ZipTip (Millipore), mixed with a matrix solution (10 mg/mL α-cyano-4-hydroxycinnamic acid, 0.1% trifluoroacetic acid, and 50% acetonitrile), and dried on a stainless steel plate. Matrix-assisted laser desorption ionization-time of flight mass spectrometry (MALDI-TOF/MS) was conducted using an ultraflex TOF/TOF (Burker Daltonics). Peptide mass fingerprinting was performed using the Mascot search program (Matrix Science).

### Polymerase activity assay.

A minigenome reporter was transfected with viral polymerase, NP expression plasmids, and firefly luciferase gene to analyze the polymerase activity (10). To determine the effect of proteins on viral polymerase activity, HEK293T or DKO cells in 24-well plates were transfected with plasmids of the PB1 (20 ng), PB2 (20 ng), PA (10 ng), and NP (40 ng), together with 40 ng minigenome reporter and 2.5 ng *Renilla* luciferase expression plasmids (pRL-TK, kindly provided by Luban) by using PEI. Cells were lysed with 100 μL of passive lysis buffer (Promega) at 24 h after transfection, and firefly and *Renilla* luciferase activities were measured using a dual-luciferase kit (Promega) with Centro XS LB 960 luminometer (Berthold Technologies). All the experiments were performed at least three times independently.

### Immunoprecipitation and Western blotting.

Cell lysates were prepared with lysis buffer (50 mM HEPES-NaOH [pH 7.9], 100 mM NaCl, 50 mM KCl, 0.25% NP-40, and 1 mM dithiothreitol [DTT]) after transfection for 24 h and centrifuged at 12,000× rpm and 4°C for 10 min. After centrifugation, 1% of cell lysates were saved as an input control, and the remaining supernatant was incubated with 5 μL of the indicated antibody plus 20 μL of protein A/G magnetic beads (MCE, HY-K0202), 15 μL of anti-Flag M2 magnetic beads (Sigma-ALDRICH, M8823), or 15 μL of anti-HA magnetic beads (MCE, HY-K0201) at 4°C for 12 h. Subsequently, the magnetic beads were collected using a magnetic bead separator and washed 5 times with phosphate-buffered saline (PBS), and 80 μL of 3× Flag peptide or HA peptide was added to each sample for elution at 4°C for 30 min or by boiling in the 1× sample loading buffer at 98°C for 10 min.

After obtaining the immunoprecipitation samples, we performed Western blotting using standard methods as described previously (6). SDS-S-polyacrylamide gel electrophoresis (PAGE) separated protein samples, which were then transferred onto nitrocellulose membranes. Membranes were blocked with 5% milk powder in Tris-buffered saline (TBS) for 2 h. Incubation with the first antibody (anti-HA antibody from Sigma [H6908], anti-Flag antibody from Sigma [F1804], anti-ANP32B from Abcam [ab200836], anti-KPNA6 from ABclonal [A7363], anti-ACTB from ABclonal [AC004], and anti-PB1, anti-NP, anti-PA, and anti-PB2 from our lab) was performed for 12 h at 4°C. The secondary antibody (Sigma, 1:20,000) was added, and membranes were incubated at room temperature for 1 h. Signals were detected using an LI-COR Odyssey imaging system (LI-COR, Lincoln, NE, USA).

### Immunofluorescence assay.

Transfected cells were washed with 1× PBS at 24 h posttransfection, followed by fixing in 4% (vol/vol) formaldehyde (Beyotime, China) for 15 min at room temperature. After incubation for 10 min with 1% Triton X-100 in 1× PBS, the cells were blocked with 5% nonfat milk in 1× PBS for 2 h. Then, the cells were incubated with primary antibodies (the mouse/rabbit anti-Flag antibody 1:300, the rabbit anti-HA antibody 1:300, the rabbit anti-KPNA6 antibody 1:100) at 4°C overnight, followed by three washes with 1× PBS. The cells were incubated with corresponding Alexa Fluor conjugated secondary antibodies (488 or 568 goat anti-mouse/rabbit IgG [H+L], Invitrogen, USA) for 1 h at room temperature. After the nucleus was stained for 10 min with 2-(4-aminophenyl)-6-indolecarbamidine dihydrochloride (DAPI), the cells were ready to have their images taken using a confocal microscope (LSM 880; Zeiss, Germany).

### RNA isolation, reverse transcription, and quantification by RT-PCR.

According to the manufacturer’s instructions, total RNA from HEK293T cells or A549 cells was extracted using the RNeasy minikit (Qiagen). A reverse transcription kit (catalog no. RR047A, Takala, China) was used for reverse transcription. A 20-μL transcription reaction system was set up with random primers and reverse transcriptase to synthesize cDNA, and 1 μg of RNA per sample was used for cDNA synthesis. SYBR Premix Ex TaqTM II (Tli RNaseH Plus) (catalog no. RR820A, TaKaRa, China) was used for real-time quantitative detection with KPNA6-specific primers (5′-ACTGTTCAGCCCTACCTTGC-3′ and 5′-CCTCCTGATGTGGCATTGGT-3′) and ACTB-specific primers (5′-AAGGAAATCTACGCCAACACG-3′ and 5′-TTTGCGGCGGACGGTAGAG-3′). The comparative cycle threshold (*C*_t_) method was used to determine the relative mRNA expression of genes normalized by the ACTB.

### Influenza virus infection.

To determine virus replication kinetics, we infected indicated cells for 1 to 2 h with each virus diluted in Opti-MEM and replaced with Opti-MEM supplemented with 0.5 to 2 μg/mL tosylsulfonyl phenylalanyl chloromethyl ketone (TPCK)-trypsin. Cell supernatants were harvested at indicated time points postinfection. The NP content in the supernatants was measured by antigen-capture enzyme-linked immunosorbent assay (AC-ELISA), and viruses were titrated using standard methods in MDCK cells as described previously (6).

### Statistics.

Statistical differences were analyzed by an unpaired two-tailed Student’s *t* test with GraphPad Prism, version 5 (Graph Pad Software, USA). All the experiments were performed at least three times independently. Error bars represent the standard deviation (SD) or the standard error of the mean (SEM) in each group, as indicated in the figure legends. The *P* values for significance are stated in the figure legends.
